# Exploration of anti-*Malassezia* potential of *Nyctanthes arbor-tristis* L. and their application to combat the infection caused by Mala s1 a novel allergen

**DOI:** 10.1186/s12906-016-1092-2

**Published:** 2016-03-31

**Authors:** Rohit K. Mishra, Vani Mishra, Anand Pandey, Amit K. Tiwari, Himanshu Pandey, Shivesh Sharma, Avinash C. Pandey, Anupam Dikshit

**Affiliations:** 1 Centre for Medical Diagnostic and Research (CMDR), Motilal Nehru National Institute of Technology (MNNIT), Allahabad, 211004 India; 2 Nanotechnology Application Centre (NAC), University of Allahabad, Allahabad, 211002 India; 3 Department of Botany, Biological Product Lab., University of Allahabad, Allahabad, 211002 India; 4 Department of Pharmaceutical Sciences, Sam Higginbottom Institute of Agriculture Technology and Sciences, Allahabad, 211007 India

**Keywords:** *Nyctanthes arbor-tristis* L. (NAT), Anti-*Malassezia* susceptibility, AUGC, Flow cytometry, Mala s1, Molecular docking

## Abstract

**Background:**

*Malassezia* commensal yeasts along with multitude of antigens have been found to be associated with various skin disorders including Pityriasis versicolor (PV). Amongst them Mala s1, a 37 kDa protein has been proved to be a major allergen reacting with a large panel of sera. However, there exists no therapeutic alternative to combat such problems in form of plant based natural compounds. The purpose of this study is in the first place, to determine the anti-*Malassezia* activity of *Nyctanthes arbor-tristis* L. (NAT) ethanolic leaf extract through turbidimetric growth curves, disruption of plasma membrane and secondly, it aims to present *in silico* validation of its active constituents over Mala s1a novel allergen.

**Methods:**

The antifungal susceptibility 50 % ethanolic extract of NAT was determined by broth microdilution method according to CLSI guidelines. Further MICs and IC_50_ were determined spectrophotometrically using the software SoftMax® Pro-5 (Molecular Devices, USA). Active constituents mediated disruption of plasma membrane was studied through flowcytometry by permeabilization of fluorescent dye Propidium Iodide (PI). Antioxidant activity of the extract was determined using the DPPH stable radical. Molecular validation of fungal DNA from the extract was observed using PCR amplification. *In silico* analysis of its active constituents over Mala s1 was performed using HEX software and visualized through Pymol.

**Results:**

The anti-*Malassezia* potential of NAT leaf extracts reflected moderate MIC 1.05 μg/μl against *M. globosa,* while least effective against *M. restricta* with MIC 1.47 μg/μl. A linear correlation coefficient *R*^*2*^ = 0.866 was obtained in case of *M. globosa* while minimum was observed in *M. restricta* with *R*^*2*^ = 0.732. The flow cytometric data reveal ~ 75 % cell death when treated with active constituents β-Sitosterol and Calceolarioside A. The docking confirmations and the interaction energies between Mala s1 and the active constituents (β-Sitosterol and Calceolarioside A) from extracts showed an effective binding which suggests Mala s1 as efficient allergen for site specific targeting.

**Conclusions:**

This study revealed that *Nyctanthes arbor-tristis* L. (NAT) extracts possess high anti-*Malassezia* potential which is driven mainly by disruption of plasma membrane. Also *in silico* validation and molecular modeling studies establishes Mala s1 as a novel allergen that could be a potential target in disease treatment. Our results would also provide a foundation for the development of new therapeutic approach using NAT extract as lead compound with high antioxidant property as an added trait for skin care.

## Background

*Malassezia* species (formerly *Pityrosporum ovale*) are exclusively the single eukaryotic member that forms a part of the resident skin microflora of human and other warm-blooded vertebrates with wide intra-species diversity [1, 2]. This intra-species diversity could be attributed to fungal evolution due to ecology, host adaptation, and pathogenicity [3]. These are opportunistic yeasts requiring predisposing environmental circumstances such as temperature and humidity, patient immune status, and genetic susceptibility [4]. *Malassezia* spp. are associated with different superficial pathogenesis, including Pityriasis versicolor (PV), Seborrheic dermatitis (SD), folliculitis as well as nosocomial bloodstream infection in pediatric care units [5, 6]. They have a species-specific ability to interact with cells and structures associated with skin, e.g. various keratinocyte subpopulations. Cell lineages involved in immune functions, including Antigen-presenting Dendritic Cells, Pattern Recognition Receptors (PRRs), Macrophages, Eosinophils and Neutrophils have also been found to be affected by *Malassezia* spp. [7, 8]. Moreover, the host interaction with *Malassezia* yeast can stimulate sensitization; elicit IgE production and T-cell reactivity. *Malassezia* extract contains a wide range of IgE binding proteins [9], however, variations in allergenic content exist. Experimental findings establish Mala s1 as a major allergen in skin disorders with 58 % IgE binding frequency [10, 11]. Mala s1 is of particular importance since it is localized in the cell wall and therefore, is easily accessible for the interplay with human innate and acquired immune system.

Many azoles derivatives are frequently used as preventive measures against fungal infections [12, 13]. However, in response to synthetic antifungal, development of resistant strains [14] and recapitulation of the disease symptoms with severe side effects have important implications in health care [15]. According to Jesus et al. [16] *Malassezia* spp. was found to be susceptible to various azole derivatives with MICs ranging from 0.01 to 4 μg/ml. However, isolates generated through in-vitro induction of resistance against fluconazole exhibited increased MICs ranging from 64 to 128 μg/ml. These strains were also found to be resistant against other azole derivatives. In recent years, natural products have been used in the discovery and development of drugs [17]. Complementary and Alternative medicines (CAM) approaches and interventions have been found to exert their effect in boosting immune response and reducing pathogen-associated symptoms [18]. In recent years they have become important molecular tools for identification of various cellular interactions because they are capable of binding specific target proteins and then interfere with their metabolism.

In India, hundreds of botanicals have traditionally been used as therapeutic alternative for majority of diseases. Many of these plants are commonly used even today. In support of this fact recently, we reported ethanolic extract of lichen *Cladia* to be effective against *Malassezia* spp. [19]. *Nyctanthes arbor-tristis* L. (NAT) commonly known as Night Jasmine (Parijatha) is one amongst them. The decoction of leaves is extensively used by Ayurvedic physicians for the treatment of arthritis, obstinate sciatica, malaria, intestinal worms and as a tonic, cholagogue and laxative [20]. In addition, analgesics, antipyretic along with ulcerogenic potency have also been observed [21]. The plant extract has also been defined to possess potent immunomodulatory activity through strong stimulation of antigen-specific and non-specific immunity as shown by increased humoral and delayed type hypersensitivity responses [22]. A water-soluble fraction of the ethanol extract elicits significant anti-inflammatory activity against acute inflammatory oedema produced in in-vivo models [23]. The 50 % ethanolic seed and root extracts of NAT also show immunomodulatory activity in systemic candidiasis infections in mice [24]. Active constituents like calceolarioside A and β-sitosterol from leaves have been reported as an efficient anti-inflammatory, anti-leishmanial and anticancer compound [25]. Thus, considering the evidence of antimicrobial and immunomodulatory potential, it would be interesting to study if the plant ethanolic extract is somehow involved in the modulation of infection caused by *Malassezia.* Anti-*Malassezia* susceptibility was studied using CLSI broth microdilution method which has recently been accepted widely [26]. Moreover, the interaction of *Malassezia* yeast with the skin results in exhaustive damage to skin baarier and its structures [27]. The dermal reconstruction is attributed to efficient synchronization of skin cells and immunomodulatory factors [28]. Accumulated research indicates effective role of antioxidant potency of botanicals in treating degenerative diseases [29, 30]. Thus, in order to address the problem of skin damage caused in PV, ethanolic NAT extract has also been assessed for its anti oxidant capacity through DPPH (1,1-Diphenyl-2-picryl-hydrazyl) assay.

Interestingly, Mala s1 has been spotted as the major allergen in the yeast and its capacity to stimulate the immune system is well documented, but its antigenicity has not yet been elucidated. Despite the fact that its 3D structure and localization in the cell wall have been elaborated, their functional details are still an enigma. Therefore, in the current study, we took a top-down approach to identify the antifungal potential of NAT leaf extract through broth microdilution assay and PCR. We systematically monitored the growth patterns of the yeast to establish the antifungal susceptibility of the plant extract. We also studied whether plant extract and the active constituents could bring about changes in cell membrane integrity by measuring Propidium Iodide (PI) influx through flow cytometer. Taking advantage of exclusive prior knowledge of NAT and its active constituents, we systematically screened two molecules by virtue of their antimicrobial ability (β-Sitosterol and Calceolarioside A) and predicted their binding on Mala s1 through molecular simulations.

## Methods

### Plant material

The fresh leaves of *Nyctanthes arbor-tristis* L. (NAT) were collected from Roxburgh garden, University of Allahabad, India. The plant was identified and authenticated from Botanical Survey of India (BSI), Allahabad and voucher specimen was kept in Duthie Herbarium, Dept. of Botany, University of Allahabad with reference number BPL/NAT/1009.

### Microorganisms

**Fig. 1:**
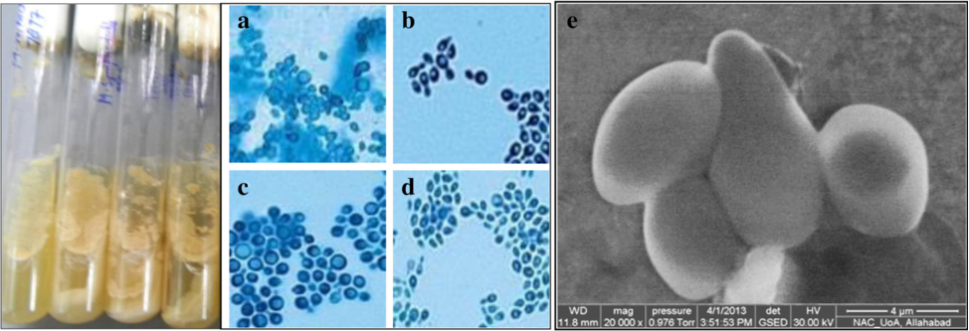
Standard strains of *Malassezia* sp. **a** *M. furfur* **b** *M. restricta* **c** *M. globosa* **d** *M. sympodialis.* Cultures were procured from Royal Netherlands Academy (Netherland) and grown in Modified Leming and Notman Agar (MLNA) and were visualized microscopically (**a** to **d**) **e** Scanning electron micrograph (SEM) of Mala s1 allergen producing *Malassezia sympodialis* (Magnification 20,000X, Pressure 0.976 Torr, WD 11.8 mm)

Four strains *M. furfur* 1878; *M. restricta* 7877; *M. globosa* 7966 and *M. sympodialis* 9974 were procured from Central Bureau voor Schimmelcultures (CBS) Fungal Biodiversity Centre, Institute of the Royal Netherlands Academy of Arts and Sciences (KNAW), Netherlands (Fig. 1).

### Preparation of ethanolic extract

Freshly collected leaves of *Nyctanthes arbor-tristis* L. (NAT) were cut into pieces and shade dried at room temperature. They were then minced using mortar. The processed sample was mixed in 50 % ethanol (1:5) for 24 h. The extracts were finally filtered and concentrated using Rotatory evaporator (Buchi type) and stored at 4 °C.

### Antifungal susceptibility test

The antifungal activity of 50 % ethanolic extract of NAT was determined using broth microdilution in 96-well flat bottom microtiter plates according to CLSI guidelines [31, 32]. Briefly, ethanolic extract of NAT was dissolved in 5–10 % DMSO to obtain a stock solution of 50 μg/μl, which was then serially diluted 1:10 in medium in order to attain final concentration ranging from 0.02 to 2.5 μg/μl. Each well was subsequently filled with 100 μl of inoculum. The initial concentration of inoculum was 1x10^6^cells/ml (adjusted according to 0.5 McFarland) in liquid media BPL5O (Patent application no. DEL/546/2012) [33, 34]. The plates were incubated at 35 ± 2 °C in wet chamber for 72 h and experiments were conducted in triplicate.

### Quantification of fungal growth

After 12 h of incubation, the optical density of the microtiter plates was recorded spectrophotometrically at 530 nm using SpectraMaxplus^384^ (Molecular Devices, USA). The process was repeated after every 12 h of incubation and continued till 72 h. The changes in OD over time were used to generate growth curves at each drug concentration against the control. The normalized OD of the extract treated wells (OD obtained after subtraction of the background OD) was used for the generation of turbidimetric growth curves. Percentage of growth inhibition at each drug concentration was calculated using the formula:

$$ \%\ \mathrm{growth} = \frac{{\mathrm{OD}}_{530}\ \mathrm{of}\ \mathrm{well}\mathrm{s}\ \mathrm{containing}\ \mathrm{the}\ \mathrm{extract}}{{\mathrm{OD}}_{530}\ \mathrm{of}\ \mathrm{the}\ \mathrm{extract}\ \mathrm{free}\ \mathrm{well}}\times 100 $$

### Area under growth curve (AUGC) analysis

AUGC was calculated and integrated using SoftMax® Pro-5 (Molecular Devices, USA). Potential differences in the OD_530_ among individual wells were accounted for by subtracting the initial OD_530_ from the values measured during the 72 h incubation, yielding net OD_530_ changes for each well. The curve was generated between AUGC vs NAT leaf extract concentration (log transformed) and a linear regression analysis was performed in MS Excel.

### Determination of minimum inhibitory concentrations (MICs)

MICs were determined spectrophotometrically using the software SoftMax® Pro-5 (Molecular Devices, USA). For the NAT extract, the MIC was determined as the lowest drug concentration showing ≥80 % growth inhibition compared with the growth in the extract-free well. Each test was performed for triplicates.

### Determination of minimum fungicidal concentration (MFC)

MFC was defined as the lowest concentration of the plant extract at which 99.99 % or more of the initial inoculum was killed. 100 μl aliquot of inoculum was taken aseptically from those wells that did not show turbidity and was poured on Modified Leming and Notman Agar (MLNA) agar plates followed by incubation for 4 days at 35 ± 2 °C. Absence of growth reflected that the concentration was cidal. The number of surviving organisms was determined by viability counts. All tests were performed for triplicates.

### Molecular validation of fungal DNA from the extract using PCR amplification

Cells from the wells were collected for DNA extraction using *AccuPrep®* Genomic DNA Extraction Kit (Bioneer, South Korea) according to the manufacturer’s protocol. PCR was performed using 4X Universal Hot Start PCR Premix (Professional Biotech India Ltd., India). The thermal cycler conditions followed were: Initial PCR activation at 95 °C for 15 min followed by 45 cycles of denaturation at 94 °C for 15 s, annealing at a 5 °C below the Tm of primers for 30s and extension of 30 s at 72 °C and final extension at 72 °C for 10 min. Primers were designed from 26S rDNA and ITS regions of all four *Malassezia* species. The primer sequences were (F) 5′ATCCTTTGCAGACGACTTGA 3′ and (R) 5′TGCTTAACTTCGCAGATCGG 3′. PCR products were run on 1.2 % agarose gel containing ethidium bromide and then viewed on Gel Documentation system (Vilber Lourmate, France) and analyzed using VISION-CAPT software (v14.3). Molecular weight marker (0.5 μg) containing a defined quantity of each band was loaded.

### Flow cytometric analysis for plasma membrane permeabilization

The membrane permeability of fungal cells after treatment with the active constituents (Calceolarioside and β Sitosterol) was measured by Propidium Iodide (PI) influx through flow cytometer. The two active constituent’s β-Sitosterol (Product No. S1 270, CAS No. 83-46-5) and Calceolarioside A (Product No. SMB00246, CAS No. 84744-28-5) were procured from Sigma-Aldrich. Cells were incubated with the two compounds at concentrations 0.31 and 0.62 μg/μl for 12 h at 35 ± 2 °C. Following incubation, cells were washed and resuspended in PBS and subsequently stained with PI (30 μg/ml) for 20 min. The cells were analyzed by BD Accuri C6 (Becton Dikinson, San Jose, CA, USA). Intrinsic parameter (SSC-A) and fluorescence in FL-2 channel for PI were acquired and recorded over logarithmic scale. The changes in treated cells were compared with untreated cells as well as with cells treated with whole NAT extract.

### Diphenylpicryl-hydrazyl (DPPH) radical scavenging capacity

The antioxidant activity of the extract was determined in terms of hydrogen donating or radical scavenging ability using the DPPH stable radical. The assay was performed according to Brad-Willam et al. [35] with slight modifications. DPPH cation solution was prepared by adding 400 μM DPPH in 0.2 M MES buffer (pH 6) and 20 % (*v/v*) Ethanol in equal volumes. 10 μg/μl of plant extract was prepared in 5–10 % of DMSO. The stock solution was further diluted to 2.5 μg/μl in 96 well Microtitre plate. DPPH cation solution was pipetted in each well and mixed with plant extract (3:1) and incubated in dark at room temperature for 20 min. Absorbance was recorded spectrophotometrically by SpectraMaxplus^384^ at 517 nm. Radical scavenging activity of the samples was expressed as IC_50_ which is the concentration of the sample required to inhibit 50 % of DPPH concentration. Ascorbic acid was taken as standard anti-oxidant with IC_50_ 0.5 μg/μl. The percent inhibition was taken into account by its FRSA count and calculated by the following formula:

$$ \%\ \mathrm{Inhibition}=\frac{\mathrm{O}.\mathrm{D}.\ \mathrm{blank}\hbox{-} \mathrm{O}.\mathrm{D}.\ \mathrm{sample}}{\mathrm{O}.\mathrm{D}.\ \mathrm{sample}}\times 100 $$

### *In-silico* analysis and molecular modeling

**Fig. 2:**
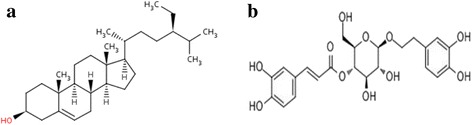
Chemical structures **a** β-Sitosterol (Pubchem ID-222284); **b** Calceolarioside A (Pubchem ID-5273566)

β-Sitosterol and Calceolarioside A were obtained from Pubchem of NCBI (http://pubchem.ncbi.nlm.nih.gov/) in SD (structural data) format and converted to 3D structure using Pc3D Molecular Viewer (Fig. 2).

Crystal structure of Mala s1 allergen of *Malassezia sympodialis* (PDB id- 2P9W) was retrieved from Protein Data Bank (www.rcsb.org). The active binding sites of the allergen were determined using Q-site identifier [36]. Around 10 sites were identified by analysing clusters of energetically favorable methyl binding sites to predict the ligand binding sites [37]. The PDB structures after molecular dynamics and simulations were submitted to HEX server [38]. The parameters used for the docking process were correlation type-shape only, calculation device- GPU, number of solutions-100, FFT mode -3D fast lite, grid dimension-0.6, receptor range-180, ligand range-180, twist range-360, distance range-40. HEX works on FFT correlation using spherical polar coordinates and Gaussian density representation of protein shape. The results were compared on the basis of docking energy and the structures were visualized through Pymol (www.pymol.org).

### Statistical analysis

All experiments were independently repeated in triplicates. Results were expressed as mean ± Standard error (SE). Linear Regression analysis was performed to determine the correlation between AUGC and drug concentration of different *Malassezia* spp. The statistical analysis was performed in MS Excel.

## Results

### In-vitro antifungal susceptibility using turbidimetric growth analysis

**Fig. 3:**
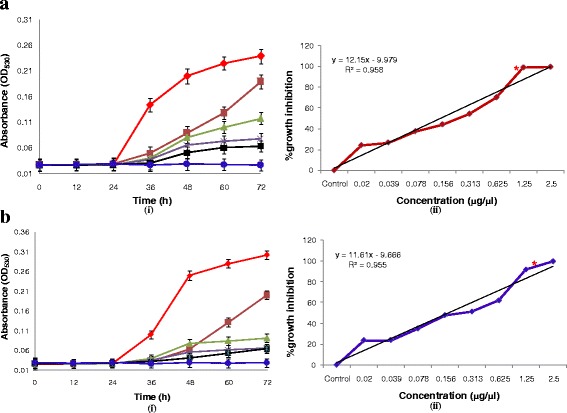
Graphical representation of the growth of **a** *M. furfur-*1878 and **b** *M. restricta-*7877 against NAT leaf extract concentrations of 0.02 (), 0.078 (), 0.313 (), 1.25 (), and 2.5 () μg/μl with control (). (**a**-**i**) and (**b**-**i**) Changes in OD_530_ over time. ODs were normalized for each growth curve by subtracting the background OD at each time point. (**a**-**ii**) and (**b**-**ii**) % growth inhibition over concentration. Data represent mean ± standard error of three replicates and * represents MIC

**Fig. 4:**
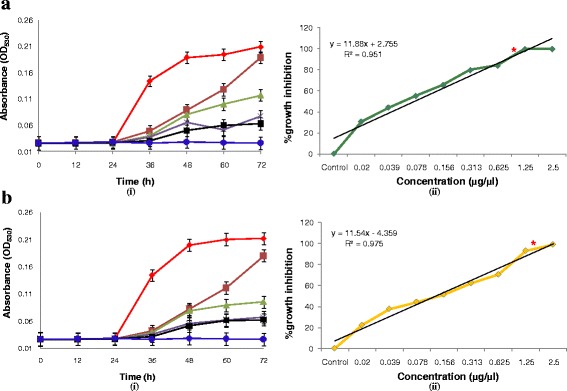
Graphical representation of the growth of **a** *M. globosa*-7966 and **b** *M. sympodialis-*9974 against NAT leaf extract concentrations of 0.02 (), 0.078 (), 0.313 (), 1.25 (), and 2.5 ()μg/μl with control (). **a**-**i** and **b**-**i** Changes in OD_530_ over time. ODs were normalized for each growth curve by subtracting the background OD at each time point. **a**-**ii** and **b**-**ii** % growth inhibition over concentration. Data represent mean ± standard error of three replicates and * represents MIC

The growth curves generated spectrophotometrically revealed variation in shape as well as growth rate with respect to extract concentrations, however, a consistent pattern was observed among the replicates. AUGC, as an important marker for pharmaco**-**dynamic study of in-vitro antifungal susceptibility, was also studied. Growth curves of *M. furfur, M. restricta, M. globosa* and *M. sympodialis* represented for 0–72 h at different concentrations of NAT extract are shown in Figs. 3a**-**i, b**-**i and 4a**-**i, b**-**i. Although, each growth phase could have been affected by each concentration in various ways (*viz*., cell wall or membrane damage, signaling blockage damage etc.), the most obvious change reflected was the shift of the growth curve towards right in response to the increasing concentration of the extract. Experiments were performed with four fungal strains over a similar dose range and conditions. Since, the results represent an undeviating pattern for all tested strains, the first result set of *M. furfur* was discussed and analyzed. The t_lag_ phase in all the growth curves stretched till 24 h that exhibited no specific changes in OD as well as AUGC values that were lower than 2 % of the maximal (data not shown) at all concentrations. However, significant changes were observed during the exponential growth phase of fungus. The control represented rapid increase in OD and about 76 % increase in AUGC was reached. The extract concentration ranging from 0.02 to 2.5 μg/μl exhibited a down regulation of OD values, thereby reducing the AUGC from 52 % for 0.02 μg/μl to 28 % for 1.25 μg/μl. The stationary phase recorded no prominent changes in OD and AUGC, thereby indicating fungal growth inhibition.

### In-vitro antifungal susceptibility for determination of MICs and MFCs

The extract showed moderate MIC of 1.05 μg/μl against *M. globosa,* while least effective against *M. restricta* with their MIC of 1.47 μg/μl (Table 1). However, ethanolic NAT extract exhibited MFC at a higher concentration (3.12 μg/μl) against all four tested strains (Fig. 1). The percent growth inhibitions of all four tested strains are expressed as Figs. 3a**-**ii, b**-**ii and 4a**-**ii, b**-**ii. A curve was plotted between extract concentration and percent growth inhibition. The graphic was complemented with a linear regression coefficient to analyze correlation between extract concentrations and percent inhibition. Significant correlation was found between extract concentrations and percent growth inhibition with a maximum *R*^*2*^ = 0.975 for *M. restricta.* Moreover, similar correlations were observed with other groups of tested strains suggesting that the data may be useful for further primary modeling of antifungal extract.

**Table 1:** Antifungal susceptibility parameters of NAT leaf extract against *Malassezia* spp. (μg/μl)

Antifungal parameters	Pityriasis versicolor (PV) causing *Malassezia* strains procured from CBS, Netherlands			
	*M. furfur* 1878	*M. restricta* 7877	*M. globosa* 7966	*M. sympodialis* 9974
MIC	1.22	1.47	1.05	1.35
IC_50_	1.17	1.11	0.74	1.31
MFC	3.12	3.12	3.12	3.12

**Fig. 5:**
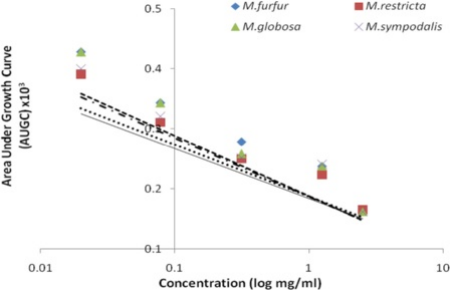
Representative linear regression (AUGC) curves for growth of *Malassezia furfur* ( *short dash*; *R*^*2*^ = 0.837), *M. restricta* ( *solid line*; *R*^*2*^ = 0.732), *M. globosa* ( *long dash*; *R*^*2*^ = 0.866) and *M. sympodialis* ( *point dash*; *R*^*2*^ = 0.804) as functions of NAT leaf extract concentration (log transformed)

The linear relationship resulted when the AUGC data were plotted as a function of antifungal extract (both log transformed) concentrations over the working range of the assay (0.02 to 2.5 μg/μl). The correlation coefficient between the AUGC and concentration of antifungal extract is expressed in Fig. 5. A maximum correlation coefficient was observed for *M. globosa* with *R*^*2*^ = 0.866 and minimum was observed in case of *M. restricta* with *R*^*2*^ = 0.732. Intermediate values were observed for *M. sympodialis* (*R*^*2*^ = 0.804) and *M. furfur* (*R*^*2*^ = 0.837). AUGC was highly correlated with the applied concentrations of extract and was found to decrease significantly with the increasing concentration. An overlapping correlation pattern was observed for all the tested strains that converged at the same point indicating that MIC values fall under the same concentration range.

### Molecular validation of fungal DNA treated with extract using PCR amplification

**Fig. 6:**
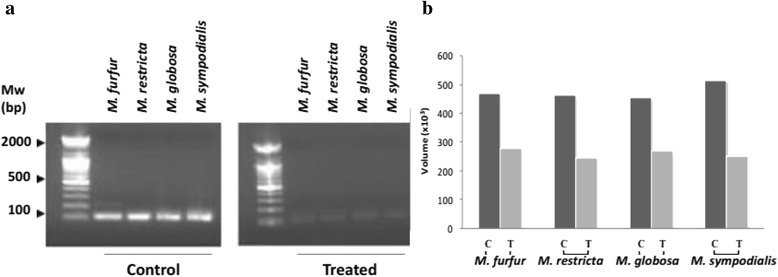
In-vitro molecular validation of antifungal susceptibility by PCR analysis. **a** Genomic DNA amplification of control and treated *Malassezia* spp. was performed using primers designed from ITS region and **b** band intensities of amplicons are presented as volume obtained from VISION-CAPT software (v14.3). MW represents 100-bp DNA ladder

The preliminary evidence of antifungal susceptibility was further validated by PCR amplification. The gel image revealed low band intensities of amplicons in treated DNA samples against comparison to the control samples. In the present study, results, therefore, reflect a good relationship between MIC and the DNA expression, suggesting that NAT extract could deplete fungal DNA thus restricting fungal growth (Fig. 6).

### Impact of active constituents on plasma membrane lesion using flowcytometry

**Fig. 7:**
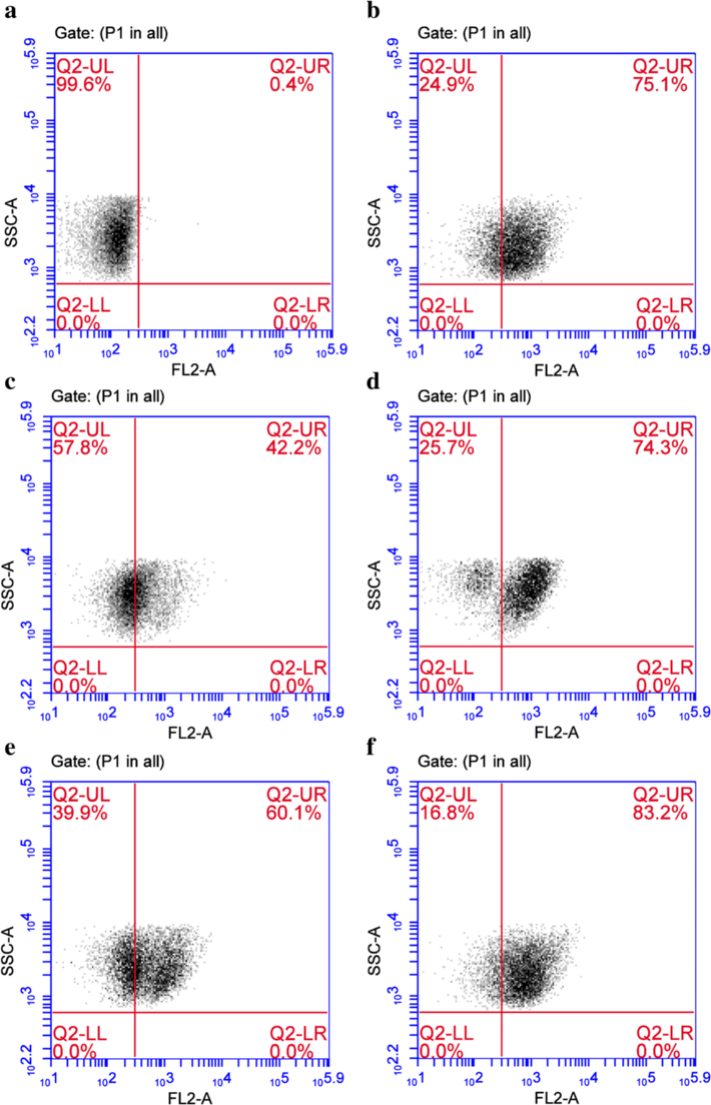
Sequence density plots showing efficacy of active constituents of NAT leaf extract on lesion of plasma membrane of *Malassezia sympodialis* with respective percentages of PI-stained cells. **a** Untreated control cells, **b** Cells treated NAT extract at MIC 1.35 μg/μl, **c** Cells treated with β-Sitosterol at 0.31 μg/μl, **d** Cells treated with β-Sitosterol at 0.62 μg/μl, **e** Cells treated with Calceolarioside A at 0.31 μg/μl, **f** Cells treated with Calceolarioside A at 0.62 μg/μl

To evaluate the effect of active constituents over plasma membrane, we measure the PI influx in cells was measured by flow cytometer. Both β-Sitosterol and Calceolarioside A treatment has shown an increase in PI influx. Figure 7a reveals no internalization of PI that represents cells with intact plasma membrane. However, a dose dependent response has been observed in active constituent treated cells. ~42.2 and 74.3 % cells have been found to show PI internalization due to lesions in plasma membrane when treated with 0.31 and 0.62 μg/μl of β-Sitosterol (Fig. 7c, d). Furthermore, similar concentrations of Calceolarioside A resulted in ~60.1 and 83.2 % cell death respectively (Fig. 7e, f). The whole NAT extract also showed ~75.1 % cell death but with a higher dose concentration that corresponds to the MIC value (1.31 μg/μl) (Fig. 7b).

### Antioxidant activity

**Fig. 8:**
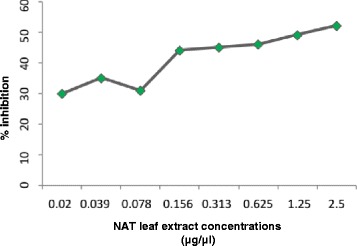
Antioxidant activity of NAT leaf extract of various dose ranges (0.02–2.5 μg/μl) was treated with DPPH cation to obtain FRSA spectrophotometrically

The leaf extract of NAT showed good antioxidant activity using Diphenylpicryl-hydrazyl (DPPH) radical scavenging capacity, which was clearly reflected in the results showing IC_50_ of 2.5 μg/μl. The reference standard used was Ascorbic acid with IC_50_ 10 μg/ml. The efficiency of plant leaf extract as potential antioxidant is an added trait (Fig. 8).

### *In-silico* molecular simulation through molecular modeling

**Fig. 9:**
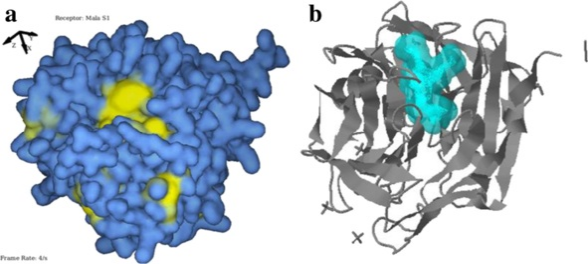
Structure and Binding sites prediction of of Mala s1. **a** The binding sites are represented in yellow color over the blue background of solid surface model of Mala s1 allergen and **b** exhibit specific binding site (*blue*) of the natural ligand in cartoon representation of Mala s1 allergen (*grey*). The sites are analyzed using Q-Site finder server predictions

In order to understand the inhibition mechanism of *Malassezia* sp. through Mala s1 allergen by natural compounds primary docking calculations were performed. The aim of the initial docking was to generate as many near native complex structures (hits) as possible. About 10 active binding sites were identified based on the interaction energy between the protein and a simple van der Waals probe. Figure 9a represents the structure and binding site of Mala s1 allergen, while Fig. 9b is the cartoon representation of the protein with Site 1 as the most favorable binding site (obtained from software server). The volume of Site 1 was estimated to be 685cubic Å and it comprises of 11 residues namely Asp39, Thr40, Ile41, Tyr42, Leu90, Ser91, Leu92, Leu93, Thr94, and His95. Following molecular dynamics simulation, the protein Mala s1 allergen with its binding site in PDB format was loaded to HEX as receptor, while the natural active constitutes found in NAT leaf extract (β-Sitosterol and Calceolarioside A) were loaded as ligands separately. Docking was performed to analyze their binding conformations. Analysis was based on Etotal or free energy of binding. For each ligand, number of hits and total energy calculations, based on shape complementarity, are listed in Table 2 for β-Sitosterol and Table 3 for Calceolarioside A.

**Table 2:** Protein-ligand docking results from HEX server, docked energies obtained from protein-ligand docking of Mala s1 allergen of *Malassezia* spp. with β-Sitosterol

Receptor protein	Ligand	Cluster	Solution	Etotal
Mala s1	β-Sitosterol	1	1	−283.1
		2	1	−239.7
		3	1	−229.8
		4	1	−223.6
		5	1	−219.7
		6	1	−216.1
		7	1	−213.2
		8	1	−210.7
		9	1	−208.4
		10	1	−206.1

**Table 3:** Protein-ligand docking results from HEX server, docked energies obtained from protein-ligand docking of Mala s1 allergen of *Malassezia* spp. with Calceolarioside A

Receptor protein	Ligand	Cluster	Solution	Etotal
Mala s1	Cal A	1	1	−354.9
		2	1	−263.3
		3	1	−252.5
		4	1	−245.0
		5	1	−240.1
		6	1	−236.1
		7	1	−232.5
		8	1	−229.5
		9	1	−227.0
		10	1	−224.7

**Fig. 10:**
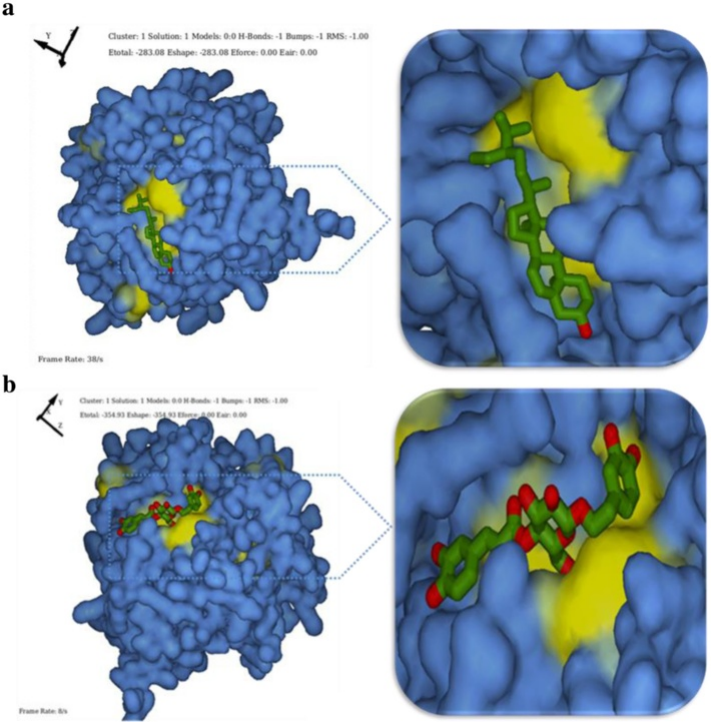
Protein-ligand docking conformation. The Protein Mala s1 allergen of *Malassezia sympodialis* is represented in blue solid surface model with yellow binding pockets. **a** The ligand β-Sitosterol represented in licorice form is embedded in the binding pocket. The binding mode as predicted by the docking program HEX (6.12v) gives an Etotal of −283.03 (*left*). The docked sites are also represented as an enlarged view (*right*). **b** The ligand Calseolarioside A represented in licorice form is embedded in the binding pocket. The binding mode as predicted by the docking program HEX (6.12v) gives an Etotal of −354.93 (*right*). The docked sites are also represented as an enlarged view (*right*)

**Fig. 11:**
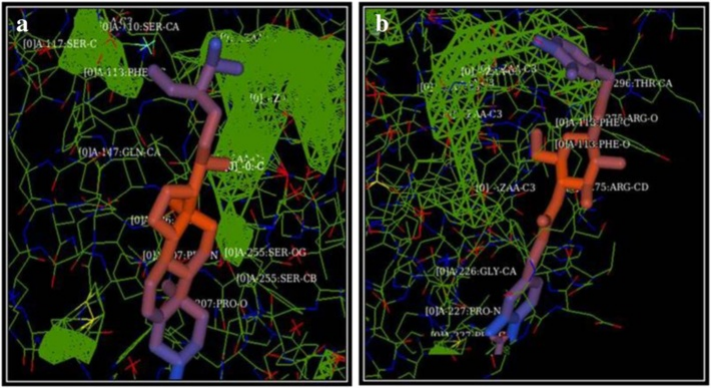
Detailed representations of binding residues of Mala s1 allergen with **a** β-Sitosterol and **b** Calceolarioside A. Mala s1 is represented in chain form with binding sites in mesh form (*green*). The residues were identified using Atom Picking mode of docking software HEX

The docking conformation of Cluster 1 of protein with β-Sitosterol showed Etotal and RMSD values of 283.1 Kcal/mol and -1Kcal/mol. Similarly, Cluster 1 of Protein-Calceolarioside rated Etotal of 354.9 Kcal/mol and RMSD of -1 kcal/mol. The docking conformations of the ligands on the binding site are represented in Fig. 10a for β-Sitosterol and Fig. 10b for Calceolarioside A. The ligands were proposed to bind at Site 1 as predicted by the Qsite finder on specific residues shown in Fig. 11.

## Discussion

Much attention has been given towards the screening of plant crude extracts and active constituents, but their mode of action has not been adequately discussed. The current study, therefore, attempts to delineate the role of NAT ethanolic extract in modulating the disease etiology caused by *Malassezia* spp. via its allergen Mala s1 through *in-silico* molecular simulations.

Since the advent of toxicology, MIC remains the hallmark of antimicrobial susceptibility testing but it does not guarantee success or failure of clinical trials. Therefore, in-vitro antifungal assay is now used to determine the development of antifungal susceptibility rather than the establishment of resistance [39]. This objective was materialized by turbidimetric measurements of fungal biomass for the generation of growth curves and other related parameters under the influence of increasing concentrations of the extract. The curves of extract free well demonstrate a lag phase (t_lag_: the period until the first detectable change in OD) of 24 h in all species indiscriminately. Similar durations of t_lag_ were observed in all tested strains indicating no inter-strain variations in growth kinetics. Moreover, it could be hypothesized that the growth patterns in different *Malassezia* spp. are regulated by a definite cluster of gene/proteins. When growth curves in the presence of increasing extract concentrations were studied, growth parameters were found to be affected as compared to extract free well. It could be documented that higher is the drug concentration, longer is the t_lag_. Similar observation was made by Chang et al. [40], while studying the growth curves based on capacitance in *Candida*. The presence of drugs decreased the germination and elongation rates of spores and germinated spores, respectively, and therefore, the critical turbidity was reached later, resulting in longer lag phases.

Since fungal growth kinetics involve long incubation times, it becomes difficult to estimate the changes in OD from the start to end point. Therefore, a calculation model based on AUGC has been developed to study even small changes of the slope, which might be an indicator of transition between phases. The log phase (t_log_) of the growth curve is that point of the curve where AUGC values increase linearly over time and the growth rate is constant at its highest value [41, 42]. AUGC represented a strong correlation with the extract concentrations (*R*^*2*^ = 0.837) in *M. furfur* with about 50 % decrease in AUGC under highest dose effect as compared to extract free well. Other species expressed results in concordance with *M. furfur*, indicating that the dose range selected was equally effective over other species under consideration and their growth kinetics in t_log_ was also in proximity.

For further determination of quality control of NAT extract, their MIC range in tested strains was established. This was accomplished using sensitive and reproducible spectrophotometrically system rather that manual visualization. The MIC for *M. furfur, M. restricta, M. globosa, and M. sympodialis* extended from 1.05 to 1.47 μg/μl. Percent growth inhibition curves indicated high correlation values with concentrations. A linear regression attributed towards strong dose dependent growth in all fungal strains. Holovachuk et al. [43] reported an inverse relationship between concentration of Gentamicin (log transformed) and bacterial growth based on AUGC. The equation used for the line was Y = A + B × log(X), where Y = AUGC, A is the y**-** intercept, B is the slope of log transformed data and X is the drug concentration. With reference to the above analysis, we established a linear relationship between fungal growth based AUGC and extract concentration (log transformed). Over the dose range of NAT extract from 0.02 to 2.5 μg/μl there existed a direct relationship between drug concentration and the growth phase. Lower extract concentration did not inhibit growth. Plotting AUGC against extract concentrations (log transformed) yielded similar findings indicating comparable working concentration range of the assay for all test pathogens.

During infection, skin bears a significant fungal load but with a distinct absence of inflammation. This down regulation of host inflammatory cascade may be attributed to the production of the fungal metabolites [44]. In such conditions the isolation and identification of the causal organisms and their molecular validation becomes an important tool for the establishment of pathogenic conditions of PV. Down regulation in band intensities of extract treated *Malassezia* cultures as compared to control indicated nucleic acid damage thereby depleting fungal population.

Earlier studies have shown that primary mechanism of action of various antifungal agents is through the disruption of plasma membranes and interfering with the normal biosynthetic pathways [45]. This attribute was studied in terms of PI influx in fungal cells treated with leaf extract as well as with two active constituents of NAT by flow cytometry. PI is a membrane-impermeabale fluorescent stain that penetrates damaged or permeabilized plasma membrane and binds to nucleic acid [46]. The whole plant extract of NAT has been found to disrupt plasma membrane and facilitate the permeabilization of PI. Also similar observations have been made with Calceolarioside B and β-Sitosterol. These results indicate that active constituents alter the cell membrane structure, causing disruption of membrane permeability barrier.

The skin diseases cause disruption of the adjoining cells and clinically results in skin fragility and blistering. Therefore, restoring and rejuvenating the skin components becomes an important aspect along with the treatment of pathogens. In this aspect antioxidants play a vital role and constitute an integral component of topical ointments. Recent studies have shown that the leaves and stem of *Nyctanthes arbor-tristis* are a potential source of natural antioxidants [47]. Ethanolic extracts of leaves and stem contains secondary metabolites of several classes *viz*., flavonoids, tannins, saponins, glycosides, alkaloids, steroids and phenolic compounds, of which phenolic compounds are also responsible for its antioxidant activity [48, 49]. The antioxidant activity in form of percent inhibition of DPPH radical showed a uniform increase with the increase in the concentration of the extract. Since inflammation and oxidative stress appear to play a significant role in Pityriasis versicolor, leaves of NAT may play an important role in wound healing through its antioxidant capacity facilitating free radicals scavenging thereby down regulating skin inflammation. Thus, the antioxidant potential marks an added advantage in the treatment of skin inflammation.

Mechanisms of action studies of natural molecules are fundamental to drug discovery. However, in-vitro studies alone may not recapitulate compound’s MoA in whole cell. Therefore, with the establishment of anti-*Malassezia* capacity of NAT in-vitro, we employed *in-silico* modeling of Mala s1 allergen of *M. sympodialis* with the bioactive constituents from NAT. Genes encoding for all 10 allergens previously cloned in different species of *Malassezia*, are now reported to exist in *M. sympodialis* genome [50]. Thus, in the current study, we addressed Mala s1 allergen of *M. sympodialis* (PDB ID: 2P9W) as a model to advance our understanding of how the yeasts interact with the host and contributes to disease pathogenesis. The localization of Mala s1 on the cell surface [51] and its role in disease etiology merits the background and frame of further investigations. Further, docking was performed between protein and active molecules (β-Sitosterol and Calceolarioside A) using Hex server and their interaction energies were analyzed (Tables 2 and 3). The low energies ensured efficient docking and also revealed that the protein could form hydrogen bond networks involving various active amino acid residues [52]. Figure 11 reveals various residues that exclusively contribute towards the binding of the protein with the natural molecules. Cumulatively, the evidence leads us to propose that the potent inhibition of cell surface allergen Mala s1 by the active constituents of NAT makes the later a unique and useful tool among the pre-existing synthetic antifungals. However, it is tempting to validate the sub-cellular machinery that will further enlighten the stage to identify new targets and their cognate inhibitors for development of novel antifungal therapeutics.

## Conclusion

The findings stated above provide an insight about the medicinal impact of NAT leaf ethanolic extract that has an inhibitory effect over *Malassezia* spp. The growth curves and the related parameters indicate that the extract can be used against different strains of *Malassezia* spp. The fungal DNA depletion associated with extract treatment also signifies the therapeutic potential of the extract. The disruption of cell membrane as observed by flow cytometry further confirms the role of active constituents as antifungal agents by driving membrane-active mechanism. Moreover, *in-silico* prediction identified specific binding sites on Mala s1 allergen that could be useful for structure-based drug designing. Further, the virtual screening of two active constituents from NAT leaf extract and their efficient docking with Mala s1 open up new avenues for the development of plant based antifungal formulations for skin care after generating toxicological data as well as successful topical testing.

## Availability of data and materials

The chemical structures of the active constituents were obtained from http://pubchem.ncbi.nlm.nih.gov/. The crystal structure of protein was obtained from http://www.rcsb.org.

## Abbreviations

AUGC: Area under growth curve
DPPH: Diphenylpicryl-hydrazyl
MFCs: Minimum fungicidal concentrations
MICs: Minimum inhibitory concentrations
NAT: *Nyctanthes arbor-tristis* L.
PI: Propidium Iodide
SE: Standard error
